# Systemic Corticosteroids And Transition To Delirium in Critically Ill Patients

**DOI:** 10.1186/2197-425X-3-S1-A30

**Published:** 2015-10-01

**Authors:** AE Wolters, DS Veldhuijzen, IJ Zaal, LM Peelen, JW Devlin, D van Dijk, AJC Slooter

**Affiliations:** University Medical Center Utrecht, Intensive Care Medicine, Utrecht, Netherlands; Leiden University, Institute of Psychology, Health, Medical and Neuropscyhology, Leiden, Netherlands; Julius Center for Health Sciences and Primary Care University Medical Center Utrecht, Department of Epidemiology, Utrecht, Netherlands; Northeastern University, School of Pharmacy, Bosten, United States

## Introduction

Delirium is frequent in the critically ill and is associated with long-term morbidity [1]. Currently, the key approach for delirium in the ICU is avoidance of risk factors. Systemic corticosteroids are often used in the ICU, and regularly administered in high dosages, as increasing evidence suggests potential benefits of these medications in critically ill patients [2, 3]. However, corticosteroids are proposed to be a risk factor delirium in patients with acute lung injury [4].

## Objective

To investigate whether systemic corticosteroids increase the probability of transitioning to delirium in a mixed medical-surgical ICU population.

## Methods

We conducted a prospective cohort study in a 32bed mixed medical-surgical ICU. Critically ill adults who stayed in the ICU for more than 24hours, without acute neurological disorders or other conditions hampering delirium assessment were included. Systemic corticosteroid administration was measured daily. Dosage were converted in milligrams (mg) prednisone equivalents. Daily mental status was classified as 'coma', 'delirium', or an 'awake without delirium' state using a previously described algorithm based on the CAM-ICU score [5]. The primary outcome, daily transition from an 'awake without delirium' state to 'delirium' was analyzed using a first order Markov multinomial logistic regression model, taking competing events such as discharge and death into account.

## Results

A total of 1112 patients accounted for 9867 ICU days. The transition from being 'awake without delirium' to 'delirium' occurred 562 times (6%). On 3483 days (35%) systemic corticosteroids were administered, with a median dosage of 50 (interquartile range 25-75) mg prednisone equivalents. Administration of systemic corticosteroids was not significantly associated with the transition to delirium (adjusted odds ratio 1.08, 95%CI 0.89-1.32). Increasing dosage on days patients received corticosteroids was not associated with an increased odds of transitioning to delirium either (adjusted odds ratio 1.00, 95%CI 0.99-1.01, per 10 mg increase in prednisone equivalents).

## Conclusions

Our findings suggest that exposure to systemic corticosteroids is not a risk factor for the transition to delirium.
